# Online consultations in mental healthcare during the COVID-19 outbreak: An international survey study on professionals' motivations and perceived barriers

**DOI:** 10.1016/j.invent.2021.100405

**Published:** 2021-05-26

**Authors:** Nele A.J. De Witte, Per Carlbring, Anne Etzelmueller, Tine Nordgreen, Maria Karekla, Lise Haddouk, Angélique Belmont, Svein Øverland, Rudy Abi-Habib, Sylvie Bernaerts, Agostino Brugnera, Angelo Compare, Aranzazu Duque, David Daniel Ebert, Jonas Eimontas, Angelos P. Kassianos, João Salgado, Andreas Schwerdtfeger, Pia Tohme, Eva Van Assche, Tom Van Daele

**Affiliations:** a Expertise Unit Psychology, Technology & Society, Thomas More University of Applied Sciences, Antwerp, Belgium; b Department of Psychology, Stockholm University, Stockholm, Sweden; c GET.ON Institute/HelloBetter, Hamburg, Germany; d Department of Clinical Psychology, Department of Clinical, Neuro-, & Developmental Psychology, Faculty of Behavioural and Movement Sciences, VU Amsterdam, Amsterdam, Netherlands; e Psychotherapy, Friedrich-Alexander University Erlangen-Nuremberg, Erlangen, Germany; f Haukeland University Hospital, University of Bergen, Bergen, Norway; g Department of Psychology, University of Cyprus, Nicosia, Cyprus; h Department of Psychology, Rouen University, Rouen, France; i Union Professionnelle des Psychologues Cliniciens Francophones et Germanophones, Belgium; j Regional Research Center for Forensic Psychiatry, St. Olavs Hospital, Trondheim, Norway; k Department of Social Sciences, School of Arts and Sciences, Lebanese American University, Beirut, Lebanon; l Department of Human and Social Sciences, University of Bergamo, Bergamo, Italy; m Universidad Internacional de Valencia, Valencia, Spain; n Cibersalud, Mallorca, Spain; o Institute of Psychology, Vilnius University, Vilnius, Lithuania; p Department of Applied Health Research, UCL, London, United Kingdom; q University Institute of Maia - ISMAI, Maia, Portugal; r Center of Psychology at University of Porto - CPUP, Porto, Portugal; s Institute of Psychology, University of Graz, Graz, Austria

**Keywords:** e-Mental health, Implementation, Telepsychology, Digital interventions, COVID-19, Online consultations

## Abstract

**Introduction:**

While the general uptake of e-mental health interventions remained low over the past years, physical distancing and lockdown measures relating to the COVID-19 pandemic created a need and demand for online consultations in only a matter of weeks.

**Objective:**

This study investigates the uptake of online consultations provided by mental health professionals during lockdown measures in the first wave of the COVID-19 pandemic in the participating countries, with a specific focus on professionals' motivations and perceived barriers regarding online consultations.

**Methods:**

An online survey on the use of online consultations was set up in March 2020. The Unified Theory of Acceptance and Use of Technology (UTAUT) guided the deductive qualitative analysis of the results.

**Results:**

In total, 2082 mental health professionals from Austria, Belgium, Cyprus, France, Germany, Italy, Lebanon, Lithuania, the Netherlands, Norway, Portugal, Spain, and Sweden were included. The results showed a high uptake of online consultations during the COVID-19 pandemic but limited previous training on this topic undergone by mental health professionals. Most professionals reported positive experiences with online consultations, but concerns about the performance of online consultations in a mental health context (e.g., in terms of relational aspects) and practical considerations (e.g., relating to privacy and security of software) appear to be major barriers that hinder implementation.

**Conclusions:**

This study provides an overview of the mental health professionals' actual needs and concerns regarding the use of online consultations in order to highlight areas of possible intervention and allow the implementation of necessary governmental, educational, and instrumental support so that online consultations can become a feasible and stable option in mental healthcare.

## Highlights

• The COVID-19 pandemic increased uptake of online consultations in mental health
• This qualitative study included 2082 mental health professionals from 13 countries.
• Mental health professionals lack previous e-mental health experience and education.
• Several barriers hinder implementation and require appropriate support.

## 1 Introduction

Mental health interventions delivered through information and communication technologies (ICT) have consistently been accumulating an evidence base over the past decades (Carlbring et al., 2018; Phillips et al., 2019). Such interventions can be labeled as e-mental health services, although numerous other terms have been proposed and the field is hampered by a lack of shared terminology (Smoktunowicz et al., 2020). Despite public interest and research support, the general uptake of e-mental health in clinical practice remains low (Irvine et al., 2020; Van Daele et al., 2020). While many mental health professionals remained skeptical or did not perceive the need for e-mental health over the past years, physical distancing and lockdown measures relating to the COVID-19 pandemic have created the demand for these services in a matter of weeks (Wind et al., 2020).

The advantages of e-mental health and blended approaches combining e-mental health and face-to-face interventions include easy access to mental healthcare, cost effectiveness, flexibility, lower stigma, and services offered in the natural context of the individual (Ebert et al., 2018; Musiat et al., 2014). Mental health professionals generally have a positive attitude toward e-mental health, but some barriers to the implementation of this technology have also been reported. The lack of knowledge on e-mental health, concerns about relational aspects, concerns about the technology itself (e.g., data security), as well as ethical, practical, and contextual factors have been suggested as hindering implementation (Mayer et al., 2019; Stallard et al., 2010). Embedding online consultations in healthcare also requires strong commitment from healthcare organizations and the support of policymakers (Shaw et al., 2018). The extent to which e-mental health is implemented in the policy and practice of mental health services varies greatly between countries. A comprehensive legal and regulatory framework, along with reimbursement schemes, is often lacking but awareness at the policy level is increasing. Some countries, such as the Netherlands and the United Kingdom, are already more advanced in the implementation of e-mental health as compared to other European countries such as Belgium and Germany (Gaebel et al., 2020). In association with the European Federation of Psychologists' Associations (EFPA) Project Group on eHealth, Van Daele et al. (2020) have recently formulated general guidelines for mental health professionals, health services, regulatory agencies, and developers to promote the development and implementation of high-quality e-mental health interventions.

Insights into mental health professionals' actual needs and concerns regarding the use of online consultations will highlight areas of possible intervention and allow for the implementation of necessary governmental, educational, and instrumental support so that online consultation can become a feasible and stable option in mental healthcare. Therefore, this study investigates the uptake of online consultations provided by mental health professionals during the first wave of the COVID-19 pandemic and aims to perform qualitative analyses to provide in-depth insights into motivations of past and current (non-)use and barriers for current use of online consultations. In this paper, online consultations are defined as an e-mental health intervention entailing digital contacts between clients and mental health professionals in the context of psychological counseling or psychotherapy, via text, audio, video, or a combination of all these. No specific hypotheses were formulated for the current study as researchers aimed to summarize the data with minimal interpretation.

## 2 Material and methods

### 2.1 Survey

In March 2020, the EFPA Project Group on eHealth set up an online survey on the use of online consultations in response to the perceived acute shift to e-mental health in and beyond Europe due to the COVID-19 pandemic. This project group was initiated in 2015 and unites experts in the field to develop a better understanding of the eHealth domain and design a sensible strategy for EFPA and its member associations. The online survey was designed to assess the extent to which mental health professionals provided online consultations at that time, their experiences with this (new) treatment modality, and their concerns. The term online consultations was not further specified and includes any digital contact between clients and mental health professionals, e.g., continuation of therapeutic sessions, but also therapist support in guided e-mental health interventions. A question on telephone consultations was also included in the survey to provide a broader picture of the shift to e-mental health in the COVID-19 pandemic, but the questions of interest for the qualitative analysis focused on online consultations. The survey consisted of 14 multiple-choice questions and 9 open-ended questions (some of which were follow-up questions that not every participant received), which could be accessed through a link on the Qualtrics platform (Appendix A). The survey was translated into 17 languages by local researchers and professionals in the field of psychology. This study focused on the qualitative analysis of mental health professionals' training in online consultations, reasons for (not) providing online consultations in the past and during the pandemic, and current barriers for the implementation of online consultations. A separate paper will utilize the quantitative survey results to model predictors of the use and experience of online consultations.

### 2.2 Recruitment

International recruitment was carried out between March 18 and May 5, 2020 through opportunity sampling via mailing lists and social media announcements of the EFPA, as well as national psychologists' associations and project collaborators from 18 countries. At this time, the participating countries imposed lockdown measures, including nationwide closure of schools and non-essential services as well as mobility limitations and physical distancing measures (all of which mandatory, except in Sweden where only the closing of upper secondary school was mandatory). The in-depth qualitative analysis relied on a subsample in which participants were included if (1) the sample from their country comprised 25 or more participants, in line with sample size recommendations for qualitative research (Guest et al., 2017; Morse, 2000), and (2) the research team's local collaborators were available to conduct a culturally sensitive analysis in the native language. A small minority of participants were excluded from the qualitative analysis because they used a language other than English or their national language(s) (e.g., Russian). In case more than 250 respondents from one country participated in the survey, a random sample of 250 participants that followed the distribution of the use of online tools of the full sample from this country was selected (Table 1). This was the case for Belgium, France, Italy, Norway, Portugal, and Sweden. This study was approved by the ethical committee of the Department of Applied Psychology of Thomas More University of Applied Sciences (Antwerp, Belgium) and informed consent was obtained from all participants.

**Table 1:** Provision of online consultations in recent days.

Country	Sample size n	Current service n (%)	Planned service n (%)	No intention to offer service n (%)
Austria	64	38 (*59.38)*	10 (*15.36)*	16 (*25.00)*
Belgium	250	167 (*66.80)*	42 (*16.80)*	41 (*16.40)*
Cyprus	45	30 (*66.67)*	6 (*13.33)*	9 (*20.00)*
France	250	103 (*41.20)*	50 (*20.00)*	97 (*38.80)*
Germany	167	83 (*49.70)*	38 (*22.75)*	46 (*27.54)*
Italy	250	194 (*77.60)*	24 (*9.60)*	32 (*12.80)*
Lebanon	73	60 (*82.19)*	8 (*10.96)*	5 (*6.85)*
Lithuania	99	62 (*62.63)*	31 (*31.31)*	6 (*6.06)*
Netherlands	81	65 (*80.25)*	13 (*16.05)*	3 (*3.70)*
Norway	250	187 (*74.80)*	28 (*11.20)*	35 (*14.00)*
Portugal	250	147 (*58.80)*	47 (*18.80)*	56 (*22.40)*
Spain	31	20 (*64.52)*	6 (*19.35)*	5 (*16.13)*
Sweden	250	119 (*47.60)*	64 (*25.60)*	67 (*26.80)*
**Total**	**2060**	**1275 (*61.89)***	**367 (*17.82)***	**418 (*20.29)***

**Fig. 1:**
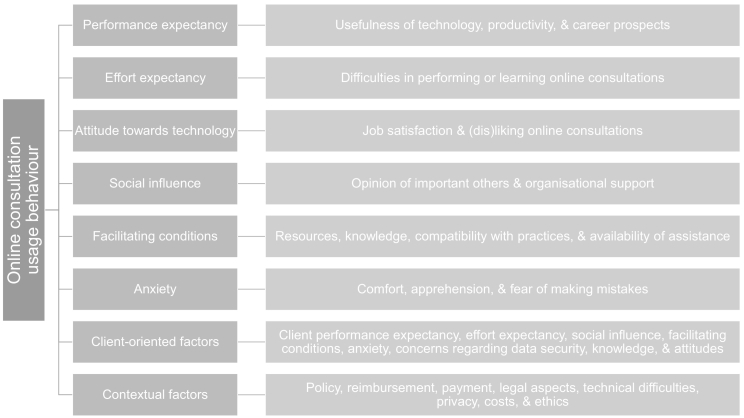
Graphical representation of the extended UTAUT-based framework of the codebook to analyze the two main open-ended questions relating to the reasons why online consultations were not used in the past and the mental health professionals' concerns regarding online consultations at that time.

### 2.3 Theoretical framework for qualitative analysis

Uptake, usage, and acceptance of technology is a multifaceted process for which several theoretical models have already been developed. Therefore, a deductive approach to qualitative analysis with a codebook, in accordance with directed content analysis (Hsieh and Shannon, 2005), was used to analyze the two main open-ended questions relating to the reasons why online consultations were not used in the past and the mental health professionals' concerns regarding online consultations at that time (Q6 and Q14 in Appendix A). To identify perceived barriers, the codebook for analysis was designed based on the Unified Theory of Acceptance and Use of Technology (UTAUT; Venkatesh et al., 2003). According to this model, technology usage behavior is determined by the intention to use, as well as facilitating conditions, including the perceived availability of technological and organizational facilities. In turn, intention to use is predicted by performance expectancy, effort expectancy, and social influence. Performance expectancy refers to whether the type of technology is expected to help in achieving goals. Effort expectancy relates to ease of use, and social influence captures whether an individual believes that important others think that they should use the technology. Other relevant factors in this framework are attitudes toward using technology, self-efficacy, and anxiety in relation to the use of technology. The UTAUT model can explain as much as 70% of the variance in the intention to use ICT (Venkatesh et al., 2003). Since participating professionals also discussed client factors in their responses, the model was extended with categories included in an adaptation of the model for end users (Ebert et al., 2015). Contextual factors and practical concerns were also included in the codebook, since the UTAUT model is mainly concerned with attitudes and beliefs. A final category of non-specific factors was incorporated, for example to account for the lack of a perceived need for online consultations altogether. Each broad category was further specified in multiple subcategories, based on the UTAUT questionnaires (Venkatesh et al., 2003; Ebert et al., 2015) and first inspection of the dataset, to provide more in-depth insights and promote clarity in the coding process.

The question on the training on online consultations (Q4_T in Appendix A) was also analyzed through deductive qualitative analysis. A coding scheme with five categories comprising a total of 17 codes was designed. These five categories were education programs focused on online consultations or e-mental health, education on online consultations as part of a different education program, informal education, knowledge based on the professionals' own experimentation, and unclear education. Further differentiation was based on the duration of training in the first category (e.g., 4 h or less), the type of education program in the second category (e.g., academic bachelor or master's in psychology), or the source of information in the third category (e.g., peer learning – intervision).

The first versions of the UTAUT-based and training codebooks were presented to all co-authors to assess clarity and piloted using small samples consisting of 10 individuals from Belgium, Lebanon, and Lithuania. The final codebook was subsequently developed through feedback and discussion with co-authors. Fig. 1 provides an overview of the categories that are represented in the final UTAUT-based codebook. The full codebook and coding instructions can be found in Table B.1 in Appendix B.

### 2.4 Analysis

Qualitative analysis based on the aforementioned codebook was performed at the national level by 14 researchers who were native-language speakers and aware of the local context of each participating country. All researchers were trained in psychology and held a PhD or were doctoral candidates. Researchers were provided with excel or SPSS sheets with anonymized data from their respective countries and additional empty variables for coding, along with coding instructions (Table B.1 in Appendix B). Any ambiguities about coding were discussed with the first author, after which consensus was reached. However, the codebook was prior developed in co-creation with the researchers, carefully piloted, and questionnaire responses were generally concise and precise. As a result, only a small minority of cases required discussion. The researchers additionally translated two open-ended multiple-choice options in which the participants could provide further input about their reasons for (not) deciding to use online consultations (Q7 and Q8 - response “other, please specify” in Appendix A). Since no coding scheme for these questions could be determined beforehand, the first author conducted inductive qualitative analysis (thematic analysis; Nowell et al., 2017) of these translated responses. An aggregated dataset was created, and frequency analyses were used to compare responses within and among countries. Distributions of the answers were visualized in frequency tables (see also B.2-B.3 in Appendix B) and country-specific as well as general findings are discussed in the results. Descriptive statistics were also calculated through frequency statistics or summary statistics for age, years of professional experience, and overall experience with online consultations (Q10, Q16, Q17 in Appendix A). The current paper focuses on the in-depth qualitative analysis in a subsample of the survey participants, a separate paper will use statistical modeling to analyze predictors of the use, the overall experience, comfort and telepresence in online consultations (including Q5, Q9, Q10 in Appendix A) in a larger sample.

## 3 Results

### 3.1 Descriptive statistics

The sample consisted of 2082 individuals, including participants from Austria (N = 65), Belgium (N = 250), Cyprus (N = 45), France (N = 250), Germany (N = 168), Italy (N = 250), Lebanon (N = 73), Lithuania (N = 119), the Netherlands (N = 81), Norway (N = 250), Portugal (N = 250), Spain (N = 31), and Sweden (N = 250). The participants had a mean age of 41.83 years (*SD* = 10.86; range: 16–80) and on average, 13.72 years of professional experience (*SD* = 9.96; range: 0–55). The survey included women (N = 1737), men (N = 336), and individuals who identified themselves as non-binary (N = 4). The majority of the included mental health professionals comprised psychologists (N = 1848), followed by psychiatrists (N = 22), mental health nurses (N = 3), or other self-specified professions (N = 206), such as psychotherapist or social worker. Most participants were self-employed (N = 859), employed in mental health organizations (N = 395), healthcare organizations (N = 355), group practices (N = 56), or other organizations (N = 413), such as educational institutions. In the Netherlands, Norway, Sweden, and to a lesser extent, Lithuania, (mental) healthcare organizations appeared to be the main employers of the participating mental health professionals.

Approximately 62% of the sample had provided online consultations in recent days, and 18% of the remaining participants intended to do so in the near future (Table 1). The survey also assessed telephone consultations, which showed a similar distribution with 1392 users, 236 planned users, and 453 non-users in recent days. France had the highest proportion (39%) of participants who were not interested in offering online consultations, while the Netherlands had the lowest (5%). The types of online consultations used in this sample were video calls (N = 1338), e-mail (N = 291), and chat (without video; N = 250). The large majority of the participants who had provided online consultations had a positive experience (n = 1111/1413), and only 94 individuals had a negative experience, resulting in a group mean score of 3.95 (*SD* = 0.82) on a 5-point Likert scale, with small differences among countries, ranging from 3.65 in Lithuania to 4.41 in Spain.

The participants who provided online consultations were asked to report the platforms they used to do so. The responses showed that many professionals used multiple platforms, depending on their clients' needs. Skype, including Skype for business, was used most often (N = 622), with the highest prevalence in Austria, Cyprus, France, Italy, Lithuania, Portugal, Spain, and Sweden (Table 2). Other frequently used platforms were ZOOM (N = 294), Whatsapp (N = 260), Whereby (N = 109), Confrere (N = 88), Microsoft teams (N = 53), FaceTime (N = 53), Facebook Messenger (N = 52), and Google services (Hangouts, Duo, Meet; N = 45).

**Table 2:** Top three most used platforms for online consultations, self-reported per country.

Country	1	N	2	N	3	N
Austria^a^	Skype	21	ZOOM	15		
Belgium	Whereby	81	ZOOM	58	Skype	56
Cyprus	Skype	24	ZOOM	6	Viber	5
France	Skype	65	Whatsapp	34	ZOOM	20
Germany	RED medical	29	ZOOM	12	Skype	10
Italy	Skype	163	Whatsapp	96	ZOOM	32
Lebanon	Whatsapp	37	Skype	31	ZOOM	13
Lithuania	Skype	60	Facebook	24	ZOOM	22
Netherlands	Quli	25	ZOOM	18	Skype	12
Norway	Confrere	86	Skype	41	Norsk Helsenett	16
Portugal	Skype	97	ZOOM	67	Whatsapp	46
Spain^a^	Skype	12	ZOOM	6		
Sweden	Skype	36	ZOOM	15	Visiba Care	13

a Platforms used by fewer than 5 individuals are excluded from this table.

### 3.2 Training in online consultations

The participants were asked to indicate whether they had received any form of training on online consultations or e-mental health and if so, to describe such training. In general, about 11% of the sample (n = 226/2082) reported having received a form of training (Table 3). Nearly half of these training programs were specific to e-mental health (n = 112/226). However, half of the e-mental health-specific training programs (N = 55) had a duration of less than 4 h. The remainder of the e-mental health-specific forms of education consisted of training with a duration of one day or less (N = 16), less than one week (N = 27), more than one week (N = 4), or a specific master's or postgraduate course (N = 6). A minority of participants had also received training in online consultations as part of a broader program, specifically in the academic training to become a psychologist in Sweden (N = 3), a professional bachelor's program in psychology in France (N = 1), a postgraduate course (Sweden: N = 1, the Netherlands: N = 2), a training school in Belgium (N = 1), or a conference workshop (Belgium: N = 1, France: N = 4, Lithuania: N = 1, Norway, N = 1). Informal education was offered through guidelines from a professional psychological organization (N = 18) or peer learning through intervision (N = 3) or supervision (N = 17). Finally, eight individuals reported having learned to use e-mental health from their own experience or experimentation.

**Table 3:** Training in online consultations.

Country	Specific training	Part of program	Informal training	Own experimentation	Unclear or unspecified	Total
Austria	9	0	1	0	5	15
Belgium	5	2	5	1	2	15
Cyprus	2	0	2	0	0	4
France	0	5	2	0	2	9
Germany	7	0	2	0	3	12
Italy	6	0	2	1	8	17
Lebanon	4	0	1	0	6	11
Lithuania	7	1	0	0	2	10
Netherlands	7	2	3	1	2	15
Norway	35	1	1	2	5	44
Portugal	6	0	13	0	5	24
Spain	3	0	0	0	2	5
Sweden	21	4	6	3	11	45
**Total**	**112**	**15**	**38**	**8**	**53**	**226**

### 3.3 Reasons for not providing online consultations in the past

Of the sample, 38% (n = 791/2078) had provided online consultations prior to the COVID-19 outbreak, with substantial differences among the countries (Table 4). Over half of the sample had previously provided online consultations in Lebanon, Spain, Cyprus, Lithuania, and Sweden, but only about a quarter of Belgian, French, and German participants had prior experience in providing online consultations.

**Table 4:** Experience with online consultations prior to the COVID-19 outbreak.

Country	Sample size n	Prior experience n (%)	No experience n (%)
Austria	65	28 (*43.08)*	37 (*56.92)*
Belgium	249	59 (*23.69)*	190 (*76.31)*
Cyprus	45	25 (*55.56)*	20 (*44.44)*
France	250	62 (*24.80)*	188 (*75.20)*
Germany	166	48 (*28.92)*	118 (*71.08)*
Italy	250	93 (*37.20)*	157 (*62.80)*
Lebanon	73	52 (*71.23)*	21 (*28.77)*
Lithuania	119	62 (*52.10)*	57 (*47.90)*
Netherlands	81	38 (*46.91)*	43 (*53.09)*
Norway	250	84 (*33.60)*	166 (*66.40)*
Portugal	249	94 (*37.75)*	155 (*62.25)*
Spain	31	18 (*58.06)*	13 (*41.94)*
Sweden	250	128 (*51.20)*	122 (*48.80)*
**Total**	**2078**	**791 (*38.07)***	**1287 (*61.93)***

The remaining participants (N = 1287) reported multiple reasons for not offering online consultations in the past (Textbox 1; Table B.2 in Appendix B). By far, the most common singular reason, reported by 33% of the individuals who had not provided online consultations in the past, was the lack of a perceived need for online consultations (n = 421/1287). The largest overall category, excluding non-specific factors, was performance expectancy. Among the professionals, 19% (n = 249/1287) were uncertain about whether online consultations were useful for their work, citing concerns about relational aspects (N = 82), using it in certain age groups, such as children (N = 35), using it with certain interventions (N = 30), working with non-verbal behavior and emotions (N = 26), using it in certain target groups or disorders (N = 20), or effectiveness (N = 20). Another commonly reported reason for not previously offering online consultations was related to the professionals' attitude, mostly disliking performing online consultations (N = 142). Problems regarding social influence were hardly related to feeling social pressure against offering online consultations (n = 3/108) but represented the lack of perceived support for online consultations by the organization or the association to which each respondent belonged (n = 103/106). In the area of facilitating conditions, the lack of resources (space and materials; n = 41/81) and the lack of knowledge (n = 33/81) were the most common reasons for not using this technology. The most common client-oriented factor that negatively influenced the implementation of online consultations was the professionals' perceived lack of client interest in using it (n = 43/70).

**Fig. 2:**
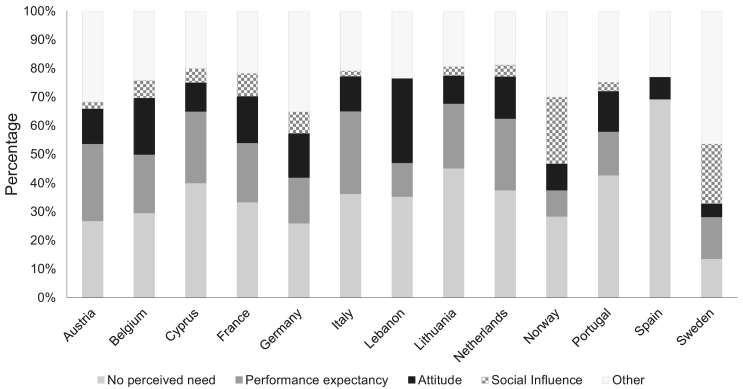
The proportions of the four most common categories, i.e., lack of perceived need, performance expectancy, attitude, and social influence, are reported relative to each country's number of participants who did not provide online consultations prior to COVID-19 (Austria: N = 37, Belgium: N = 190, Cyprus: N = 20, France: N = 188, Germany: N = 118, Italy: N = 157, Lebanon: N = 21, Lithuania: N = 57, Netherlands: N = 43, Norway: N = 166, Portugal: N = 155, Spain: N = 13, and Sweden: N = 122).

The countries showed some differences in the most common reasons for not offering online consultations in the past (Fig. 2). The lack of a perceived need was cited by the largest subgroup of previous non-users in all countries except Sweden, where facilitating conditions (mostly the lack of resources) comprised the most commonly reported category. In Spain, the perceived need for online consultations was very low, and performance expectancy and social influence were not reported; however, the interpretation of these findings is hampered by the small sample size (13 participants without previous use out of a total of 31 Spanish participants). Factors relating to social influence, specifically the lack of perceived support from the participants' organizations or associations, were more regularly reported in Sweden (N = 23) and Norway (N = 28) compared with the other participating countries.

Textbox 1 **Most frequently reported reasons for not providing online consultations prior to the COVID-19 outbreak, both as singular coded responses and in the form of categories of the UTAUT-based coding scheme. Non-specific factors are not included in most common broader categories.**

**Table:** 

**Most reported singular reasons** 1. I did not or do not have a need for online consultations (non-specific factors; N = 421). 2. I do not like doing online consultations (compared with face-to-face sessions) (attitude toward using technology; N = 142). 3. My organization or association has not provided sufficient support for online consultations (social influence; N = 103). 4. I have concerns about relational aspects (e.g., impersonal contact, fostering a therapeutic relationship) (performance expectancy; N = 82). 5. Clients are not interested in using online consultations (client-oriented factors - attitudes; N = 43). **Most common broader categories** 1. Performance expectancy (N = 249) 2. Attitude toward using technology (N = 181) 3. Social influence (N = 106) 4. Facilitating conditions (N = 81) 5. Client-oriented factors (N = 70)

Alt-text: Textbox 1

### 3.4 Reasons for (not) providing online consultations during the pandemic

When answering a multiple-choice question, the mental health professionals indicated multiple reasons why they decided to start providing online consultations at present or in the near future. Among the participants, 75% (n = 1237/1642) considered online consultations a necessity from a public health perspective, 69% (n = 1139/1642) wanted to serve and support their clients who could not attend face-to-face sessions, 35% (n = 576/1642) reported that their clients wanted it, 31% (n = 505/1642) wanted to stay in touch with new developments in technology, and another 30% (n = 491/1642) did not want to lose income. Among the participants, 9% (n = 148/1642) provided additional self-specified reasons; the most common ones include the following: it was necessary due to the pandemic and the related lockdown and quarantine measures (N = 44); online consultations were required by their organization, association, or government (N = 33); they wanted to continue the therapeutic process and care (N = 17); and they were already conducting online consultations before the pandemic (N = 16; mostly to overcome distance barriers with clients who were living far away, N = 11).

The mental health professionals who had not provided online consultations during the first month of the outbreak (N = 418) selected the following UTAUT-based reasons for this in a multiple choice question: online consultations do not seem as effective as face-to-face consultations (performance expectancy; N = 129); I lack the required hardware or software (facilitating conditions; N = 129); my clients do not want this (client attitude; N = 83); I do not know how to use it in practice (facilitating conditions; N = 56); I generally dislike using technology in practice (attitude; N = 55); I currently do not see the value over continuing face-to-face (performance expectancy; N = 43); technology is unreliable (contextual factors; N = 36); I am afraid to make mistakes (anxiety; N = 23); it requires too much effort (effort expectancy; N = 18); my colleagues disapprove (social influence; N = 3); or another self-specified reason (N = 81). The most reported additional reasons were the following: their work context did not allow online consultations (N = 22); they were not seeing patients (N = 13); and they were concerned about privacy issues (N = 10).

### 3.5 Perceived barriers for current use of online consultations

**Fig. 3:**
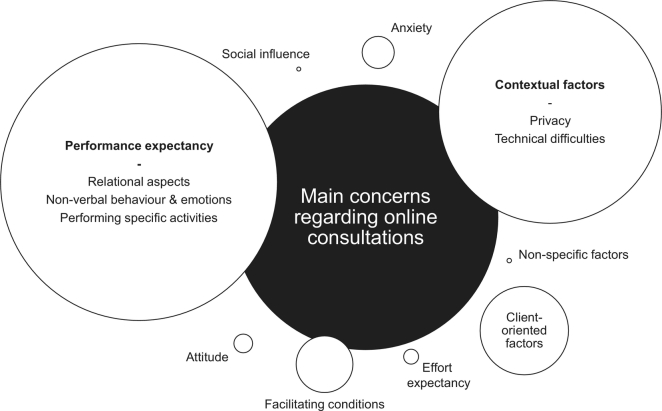
Visual overview of the results of the qualitative analysis on the main concerns or questions professionals had regarding online consultations during the first wave of the COVID-19 pandemic. The size of the spheres is proportional to the number of concerns that were reported in each category. Further specifications are included for the two largest categories.

Fig. 3 provides an overview of the concerns of professionals regarding online consultations. A total of 1420 participants reported one or more concerns regarding the current use of online consultations (Table 5). A detailed report of regional responses can be found in Table B.3 in Appendix B.

#### 3.5.1 Performance expectancy

Performance expectancy was the largest category of concerns. The participants in all countries clearly had several concerns about whether online consultations would be useful for their work. Approximately 17% of the entire sample (n = 357/2082) were worried about relational aspects of online consultations, which could include fostering a therapeutic relationship, the lack of eye contact and physical presence, and the lack of authentic contact. Other common themes, reported by over 10% of the entire sample, involved how to work with non-verbal behavior and emotions (n = 215/2082) and how to carry out certain diagnostic assessments or interventions (n = 231/2082; e.g., exercises or specific therapeutic interventions, such as exposure therapy and eye movement desensitization and reprocessing). Professionals further reported concerns about using online consultations with specific populations (N = 126), such as individuals who experienced trauma, and age groups (N = 111), such as children. A limited number of participants had concerns about effectiveness (N = 74). A minority of participants (N = 11) reported lower productivity due to online consultations, and only one noted a negative influence of online consultations on his/her career.

**Table 5:** Overview of the number of concerns (per country) in the different categories.

Country	PE^a^	EE^b^	AT^c^	SI^d^	FC^e^	AN^f^	COF^g^	CF^h^	NF^i^
Austria	43	1	9	0	5	6	11	55	4
Belgium	159	23	7	0	35	29	65	147	3
Cyprus	29	0	2	2	3	6	8	22	0
France	119	2	3	2	54	7	33	127	0
Germany	97	2	8	0	10	15	40	97	1
Italy	134	6	5	0	10	12	40	88	0
Lebanon	30	0	1	0	5	4	11	40	0
Lithuania	89	3	1	2	32	4	27	71	0
Netherlands	91	10	13	0	7	7	24	34	0
Norway	116	1	7	7	17	14	40	67	1
Portugal	164	6	15	0	23	13	45	109	0
Spain	4	2	2	1	3	0	3	9	0
Sweden	96	7	6	3	36	19	27	76	10
**Total**	**1171**	**63**	**79**	**17**	**240**	**136**	**374**	**942**	**19**

a PE: performance expectancy.

b EE: effort expectancy.

c AT: attitude toward online consultations.

d SI: social influence.

e FC: facilitating conditions.

f AN: Anxiety.

g COF: client-oriented factors.

h CF: contextual factors.

i NF: non-specific factors.

Concerns about performance expectancy were common in all countries and especially prominent in the Netherlands and Lithuania. Dutch participants were particularly concerned about executing certain interventions (n = 23/81) and working with non-verbal behavior and emotions (n = 21/81). Lithuanian participants were more concerned about relational aspects (n = 23/119) and using online consultations with clients with certain disorders or target groups (n = 22/119).

#### 3.5.2 Effort expectancy

The mental health professionals reported a limited number of concerns about the amount of effort required in online consultations. A minority of participants reported difficulties in performing online consultations (N = 31), found online consultations more exhausting (N = 27), or struggled with learning to use the technology (N = 5). However, it is relevant in this regards that most professionals used common online communication software (e.g., Skype) as opposed to specialized platforms for online therapy, which might require more effort and technological competencies. In Cyprus and Lebanon, no concerns were raised regarding effort expectancy, in contrast to Belgium (N = 23) and, to a lesser extent, the Netherlands (N = 10).

#### 3.5.3 Attitude

Disliking online consultations or preferring to work face-to-face was not a main concern for the professionals (N = 12). While a small number of participants reported missing closeness, contact, and an authentic meeting (N = 64), only 3 indicated that online consultations made their job less interesting.

#### 3.5.4 Social influence

A minority of cases reported the lack of support from their organizations or associations as their current main concern (N = 16), and only one individual noted unnecessary prejudice from clients and colleagues.

#### 3.5.5 Facilitating conditions

Concerns about lacking the necessary resources for online consultations were common and mainly involved lacking knowledge about or wanting more education about online consultation (N = 148). Furthermore, a small group of participants lacked materials or undisturbed space to do online consultations (N = 60). A minority of participants voiced concerns about incompatibility of online consultation software with other systems or practices (N = 27) or about lacking support in terms of assistance with system difficulties (N = 5). The reported need for more education was greater in France (n = 48/250) and Lithuania (n = 22/119) than in Norway (n = 5/250), Italy (n = 7/250), and Lebanon (n = 2/73).

#### 3.5.6 Anxiety

A limited number of participants reported concerns about feeling apprehensive toward online consultations (N = 80). However, this was mostly due to some therapists' fear of loss of privacy (e.g., sharing a Skype number, patients recording the session; N = 48) and to a lesser extent, to some professionals feeling uncomfortable with doing online consultations (N = 32). The participants who reported their fear of making mistakes that could not be corrected (N = 50) were mostly afraid of experiencing technical difficulties (N = 33). Online consultations were generally not considered as intimidating (N = 6). Apprehensions about online consultations were mainly noted in Belgium (n = 20/250), and the fear of making mistakes was most common in Sweden (n = 15/250).

#### 3.5.7 Client-oriented factors

Mental health professionals also raised concerns about potential problems with the implementation of online consultations on the client side. They were concerned about facilitating conditions for their clients (N = 230), including clients' lack of the necessary technical possibilities or undisturbed quiet space (N = 193) and to a lesser extent, lack of technical knowledge (N = 33) or support (N = 4). A smaller number of the respondents raised other client-related concerns, such as clients feeling apprehensive about or uncomfortable with online consultations (N = 52) and the lack of client interest (N = 47; with the highest rate in Belgium (n = 14/250)). A small number of concerns were raised regarding their clients' own issues: performance expectancy (N = 16), concerns regarding data security (N = 14), effort expectancy (N = 7), knowledge about online consultations (N = 7), and social influence (N = 1).

#### 3.5.8 Contextual factors

The concern that was raised most often in the survey, by over 20% of the entire sample, involved the privacy and security of online consultation software (n = 442/2082), especially in Austria (n = 28/65), Lithuania (n = 36/119), and Germany (n = 46/166), as opposed to Norway (n = 31/250), Sweden (n = 36/250), and Lebanon (n = 10/73). The second concern was related to unreliable connectivity and technical difficulties (N = 261). A number of professionals asked other practical questions about charging and management of payments (N = 88; especially in France, n = 23/250), the limits of responsibility and legal aspects (N = 50), ethical standards (N = 38), policy and administration (N = 36), the price of high-quality platforms (N = 16), and reimbursement and insurance (N = 11).

## 4 Discussion

Mental health professionals quickly and flexibly adopted online consultations in the beginning of the first wave of the COVID-19 pandemic. The majority of them had positive experiences with this mode of delivery, and it seems that online consultations have the potential to become more than just temporary replacements of face-to-face consultations in times of crisis. This study provides an overview of the factors that can hinder implementation with the goal of promoting the provision of the necessary support for the deployment of online consultations and other e-mental health interventions.

While the lack of the need for online consultations was the most important reason for not implementing them in the past, this need has become strong and acute due to the COVID-19 pandemic. Several other barriers to using online consultations have nevertheless remained. Mental health professionals still share concerns about whether online consultations are useful for their work, for example, concerning relational aspects, working with non-verbal behavior and emotions, performing certain assessments or interventions, or working with certain populations. Such concerns are not new (e.g., Stallard et al., 2010), but accumulating evidence shows the relevance of the therapeutic relationship in e-mental health (Kaiser et al., 2021) and suggests the equivalence of relational aspects in different modes of delivery (Irvine et al., 2020). Online consultations also appear feasible across different diagnostic groups and capable of reaching similar clinical outcomes as compared to conventional treatment (Chiauzzi et al., 2020). This includes individuals with serious mental illness, although extra care and consideration is warranted for individuals with elevated suicide risk. As noted in the survey, the professionals also had practical concerns about the privacy and security of online consultation software and experiencing technical difficulties, as well as about clients having the necessary technical possibilities or undisturbed quiet space. This study indicates that internal factors, such as the professionals' attitudes or fears regarding online consultations, did not have a great influence during the first wave of the COVID-19 pandemic. However, the mental health professionals expressed a clear need for knowledge on psychological processes in online consultations, as well as technical implementation aspects.

These perceived barriers are in line with the mental health professionals' lack of pertinent education in online consultations or e-mental health, especially in France, Belgium, Italy, and Germany. The training received by the participants consisted mostly of a session of a few hours. The COVID-19 pandemic did result in several short-term local and international initiatives, providing training in online consultations for professionals through brief webinars. Many psychologists' associations and the EFPA (European Federation of Psychologists' Associations, n.d.) also provided guidelines for the implementation of online consultations. Nevertheless, even in the countries with the highest reported rates of education, i.e., Austria, the Netherlands, and Sweden, still over 75 to 80% of mental health professionals did not receive any education. Centralized international initiatives that outline institutional training requirements in order to use virtual care services and promote common standards in e-mental health education programs, good practice examples of online consultations, and information on how to deal with ethical concerns and confidentiality issues (of communication software) are necessary. We need to consider devising future guidelines on these topics for Europe, knowing that guidelines for telepsychology have existed since 2013 in the USA (Joint Task Force for the Development of Telepsychology Guidelines for Psychologists, 2013).

There were cross-national differences in uptake and perceived barriers for the implementation of online consultations. Over a quarter of mental health professionals in France and Germany did not intend to implement online consultations. These countries, together with Belgium, also show the lowest rates of previous use. On the other hand, in Lithuania, the Netherlands and Lebanon (the only participating Arab country), mental health professionals have a higher current uptake and more existing experience in delivering online consultations. While mental health problems carry a lot of stigma in Arab countries (Househ et al., 2019), which could increase interest in more anonymous e-mental health contacts, mental health legislation and infrastructure (including telepsychiatry) is often still underdeveloped in these countries, including Lebanon (El Hayek et al., 2020). A considerable number of Lebanese mental health professionals have received at least a portion of their training outside the Arab countries' borders, which could imply that they are more culturally “close” to the western societies.

Gaebel et al. (2020) have shown that European countries are in varying stages of implementing e-mental health in their mental healthcare systems. Some differences among the countries in this study can be associated with different regulations and “maturity” in the eHealth domain. For example, both Norway and Sweden have a reimbursement scheme for digital interventions, as well as its national health authorities' guidelines on which platforms to use. The Netherlands also have a regulatory framework for online consultations. In other countries, such as Germany and Belgium, governments have provided a temporary framework and guidelines within which mental health professionals could operate for the duration of the COVID-19 pandemic. Results indeed show that mental health professionals from Norway, the Netherlands, and Germany use more specialized platforms for online consultations. However, Sweden showed a lot of variation in the platforms used, including several organization-specific tools. Germany has a list of tools that professionals are allowed to use within the legal framework (adjusted due to the COVID-19 pandemic), and the use of other tools, such as Skype, is not in line with the country's regulations. In Spain, the Guide for Telepsychological Intervention (De la Torre and Pardo Cebrián, 2018) is a first reference document of the psychotherapeutic online approach and the Official College of Psychologists of Madrid provides free access to a platform for online psychotherapy to affiliated members. While regulating digital health applications holds many challenges, innovative approaches engaging policy makers, developers, and patients and professionals have already been suggested (Rodriguez-Villa and Torous, 2019).

This study has several limitations. Qualitative analyses were guided by a theoretical framework and executed in a uniform way, but different local researchers performed the coding for the different countries with varying sample sizes. While local researchers were aware of the national context in terms of culture and policy, having just one rater precluded the calculation of interrater agreement and reliability. It also leaves room for subjective interpretation in the inductive analysis, however, given that the thematic analysis was only performed on brief and concrete survey responses (e.g., “due to COVID-19”), the potential for rater bias was limited. While similar lockdown measures were implemented in all countries during the 39-day recruitment period, concerns regarding online consultations and other questionnaire responses could potentially vary depending on the exact moment of questionnaire completion. Considering the survey was disseminated and completed online, a potentially biased sample toward individuals who were already fairly comfortable with the use of online tools cannot be ruled out. Individuals who were disinclined to use online tools were likely underrepresented in the results. The sample of mental healthcare professionals mostly consisted of psychologists. We did not differentiate between types of online consultations while experiences and perceived barriers could vary depending on implementation characteristics. While online consultations could be a part of a guided self-help intervention, the vast majority of online consultations are expected to have taken place in the context of traditional therapeutic contacts, given the acute shift online due to the pandemic. Future research should differentiate between types of online consultations and would benefit from a common glossary regarding digital psychological interventions (Smoktunowicz et al., 2020). The clients' concerns, beliefs, and practical requirements should also be assessed first-hand.

To conclude, for some mental health professionals, the current crisis will prove to be a turning point that will lead to an increased use of digital tools in practice. However, other professionals have difficulty in finding their way, perceive that online consultations do not meet their or their clients' needs, or work in a context that does not easily lend itself to online consultations. Moving forward from the acute threats that the COVID-19 pandemic poses to mental health practice, policymakers and practitioners should aim for a selective implementation of high-quality e-mental health interventions by professionals who have received sufficient training. However, as Shaw et al. (2018; p95) state, “mainstreaming virtual consulting across multiple departments in multiple organizations will be neither smooth nor quick. The clinical and logistical realities will play out differently for different clinical specialties and different hospital departments (not to mention primary care).” This study shows a clear need for training in online consultations that is shared by different countries and provides further insights into the barriers to high-quality implementation of online consultations and e-mental health. These factors should be considered when healthcare organizations and local, national, and European governmental agencies set up long-term strategic goals and implementation roadmaps for the future.

The following are the supplementary data related to this article.Appendix A Online survey that was used to collect the data. Appendix A

Appendix B Presentation of UTAUT-based codebook and full tables with the results of the qualitative analysis of the open-ended questions on the reasons for past non-use of online consultations and current concerns regarding online consultations. Appendix B

## Declaration of competing interest

The authors declare the following financial interests/personal relationships which may be considered as potential competing interests:

Assoc. Prof. Ebert reports to have received consultancy fees or served in the scientific advisory board from several companies such as Minddistrict, Sanofi, Lantern, Schön Kliniken, German health insurance companies (BARMER and Techniker Krankenkasse), and chambers of psychotherapists. Dr. Ebert is one of the stakeholders of the Institute for health trainings online (GET.ON/HelloBetter), which aims to implement scientific findings related to digital health interventions into routine care. Anne Etzelmueller is employed by the Institute for health trainings online (GET.ON/HelloBetter) as research coordinator. All other authors do not report any conflict of interest.
