# Supplementary material for: Online consultations in mental healthcare during the COVID-19 outbreak: An international survey study on professionals' motivations and perceived barriers
Source: Internet Interv. 2021 May 26;25:100405. doi: 10.1016/j.invent.2021.100405 (PMC8350604; doi:10.1016/j.invent.2021.100405)
Supplement: The following are the supplementary data related to this article.Appendix A — Online survey that was used to collect the data. [file mmc1.docx]

Appendix 1

Online survey that was used to collect the data

Online consultations for mental healthcare

*Choose your language at the top.*

**Goal and methodology of this study**
This brief anonymous questionnaire from the [EFPA Project Group on eHealth](http://ehealth.efpa.eu) takes about 5 minutes to complete and focuses on the current use of online consultation technology by psychologists and other mental healthcare professionals in the context of the COVID-19 Coronavirus pandemic.

**The aim is to obtain an overview of your current usage and your main questions and concerns for adequately using online consultation. This questionnaire should not be used for urgent questions that require personalised answers. In case of such questions, reach out to your professional association, national authority or national government!** We want your input to inform national stakeholders across Europe and beyond and to use this data to further research in this domain. Results will be globally communicated by EFPA, the European Federation of Psychologists' Associations. National associations or governments who want to obtain the data specifically for their country, are encouraged to reach out to us.

- I understand what is expected of me during this study.
- I myself, or others, may benefit from this research as it will help to inform current and future practice and address current needs, questions and concerns on online consultation.
- I understand that my participation in this study is voluntary. I have the right to discontinue my participation at any time. I don't have to give a reason for that and I know that it won't be a disadvantage for me.
- The results of this study may be used for scientific purposes and may be published.
- My name will not be published, anonymity and the confidentiality of the data is guaranteed at every stage of the research in accordance with international guidelines (ICH-GCP), [including GDPR](https://www.thomasmore.be/en/privacy-statement).
- This study has been approved by the ethical committee of [the Department of Applied Psychology of Thomas More University of Applied Sciences (Antwerp, Belgium)](http://mailto:ethischecommissie.tp@thomasmore.be)
- For questions after my participation, I know that I can contact the supervisor of this study: Tom Van Daele
   on behalf of the EFPA Project Group on eHealth
   [tom.vandaele@ehealth-psychology.eu](mailto:tom.vandaele@ehealth-psychology.eu?subject=Online%20consultation%20for%20mental%20healthcare%20-%20questionnaire)
- I have read and understood all of the information above and I agree to participate in this study.

Q1 I have made use of consultations over the telephone in recent days.

- yes
- no, but I intend to do so in the near future
- no

Q2 I have made use of online consultations in recent days.

- yes
- no, but I intend to do so in the near future
- no

Q3 I have experience with online consultations, prior to the COVID-19 outbreak.

- yes
- no

Q4 Have you had any specific training concerning online consultations?

- yes
- no

Display This Question:

If Have you had any specific training concerning online consultations? = yes

Q4_T Could you please describe this? 
*Title and number of hours*

________________________________________________________________

________________________________________________________________

Q5 To what extent do you consider yourself to be comfortable with (the concept of) online consultations?

- Highly uncomfortable
- Somewhat uncomfortable
- Neutral
- Somewhat comfortable
- Highly comfortable

Display This Question:

If I have experience with online consultations, prior to the COVID-19 outbreak. = no

Q6 Is there any particular reason why you did not make use of online consultations in the past?

________________________________________________________________

________________________________________________________________

Display This Question:

If I have made use of online consultations in recent days. = yes

Or I have made use of online consultations in recent days. = no, but I intend to do so in the near future

Q7 What is/are your reasons for starting to make use of online consultations at the moment?

- I consider it a necessity from a public health point of view
- My clients want this
- I do not want to lose income
- I want to be there for clients who cannot attend.
- I want to stay in touch with how technology use of clients is currently evolving
- Other, please specify ________________________________________________

Display This Question:

If I have made use of online consultations in recent days. = no

Q8 What is/are your reason(s) for not starting to make use of online consultations at the moment?

- It does not seem to be as effective as face to face
- I currently do not see the added value over continuing face to face
- It is too much effort
- I generally dislike using technology in practice
- My colleagues disapprove
- My clients do not want this
- I do not know how to use it in practice
- I lack required hard- or software
- Technology is unreliable
- I am afraid to make mistakes
- Other, please specify ________________________________________________

Display This Question:

If I have experience with online consultations, prior to the COVID-19 outbreak. = yes

Or I have made use of online consultations in recent days. = yes

Q9 If you started online consultations recently, how would you qualify your level of telepresence (feeling of being connected with one another) during consultations?

- Very low
- Somewhat low
- Neutral
- Somewhat high
- Very high

Display This Question:

If I have experience with online consultations, prior to the COVID-19 outbreak. = yes

Or I have made use of online consultations in recent days. = yes

Q10 I would describe my overall experience with online consultations as follows

- Highly negative
- Somewhat negative
- Neutral
- Somewhat positive
- Highly positive

Display This Question:

If I have experience with online consultations, prior to the COVID-19 outbreak. = yes

Or I have made use of online consultations in recent days. = yes

Q10_T If you want to, please elaborate:

________________________________________________________________

________________________________________________________________

Display This Question:

If I have experience with online consultations, prior to the COVID-19 outbreak. = yes

Or I have made use of online consultations in recent days. = yes

Q11 For online consultations I make use of

*multiple answers possible*

- chat (without video)
- video calls
- email
- other, please specify ________________________________________________

Display This Question:

If I have experience with online consultations, prior to the COVID-19 outbreak. = yes

Or I have made use of online consultations in recent days. = yes

Q12 On which software platform(s) do you rely for online consultations?

________________________________________________________________

________________________________________________________________

Display This Question:

If I have made use of online consultations in recent days. = no, but I intend to do so in the near future

Q13 On which software platform(s) do you intend to rely for online consultations?

________________________________________________________________

________________________________________________________________

Q14 The main concerns or questions regarding online consultations I currently have are the following. 
*Don’t hesitate to write down more than one. If you don’t have any experience, write down what would help to consider / starting to make use of it. If you already rely on online consultation, write down questions that might have arisen, or concerns which you would like to raise based on your experience.*

- 1 ________________________________________________
- 2 ________________________________________________
- 3 ________________________________________________
- 4 ________________________________________________

Q15 Profession

- Psychologist
- Psychiatrist
- Mental health nurse
- Other, please specify ________________________________________________

Q16 Age (in years, please write down a number)

________________________________________________________________

Q17 Professional experience (in years, please write down a number)

________________________________________________________________

Q18 Professional situation
*indicate your main profession*

- Self employed
- Group practice
- Healthcare organization
- Mental healthcare organization
- Other, please specify _______________________________________________

Q19 Gender

- Male (M)
- Female (F)
- Non-binary (X)

Q20 Country of residence

▼ Afghanistan (1) ... Zimbabwe (1357)

Q21 Local province, region or state

________________________________________________________________

Q22 Additional comments: if there is anything else you would like to share, you can do so here.

________________________________________________________________

________________________________________________________________

________________________________________________________________
