# Supplementary material for: Online consultations in mental healthcare during the COVID-19 outbreak: An international survey study on professionals' motivations and perceived barriers
Source: Internet Interv. 2021 May 26;25:100405. doi: 10.1016/j.invent.2021.100405 (PMC8350604; doi:10.1016/j.invent.2021.100405)
Supplement: Appendix B — Presentation of UTAUT-based codebook and full tables with the results of the qualitative analysis of the open-ended questions on the reasons for past non-use of online consultations and current concerns regarding online consultations. [file mmc2.docx]

Table B.1

UTAUT-based codebook for the analysis of the open-ended questions on the reasons for not using online consultations in the past and the concerns individuals had regarding online consultations. Coders received the instructions to “Read through the categories of the codebook so you have a good understanding of the possible answers. For Q6 and Q14 Add ‘1’ in the respective column, multiple categories can be present simultaneously and should all be registered”.

| **Topic** | **Category** | **Nr.** | **Subcategory** |
| --- | --- | --- | --- |
| mental health professional factors | 1. Performance expectancy | 1.1. | I have questions about whether online consultations are useful in my job (general) |
|  |  | 1.1.1. | Useful in my job: Concerns about effectiveness |
|  |  | 1.1.2. | Useful in my job: Concerns about how to use it with certain age groups (children, elderly) |
|  |  | 1.1.3. | Useful in my job: Concerns about how to use it with certain disorders/target groups |
|  |  | 1.1.4. | Useful in my job: Concerns about how to do certain interventions (e.g., exercises, exposure therapy, EMDR,…) or diagnostics |
|  |  | 1.1.5. | Useful in my job: Concerns about relational aspects (e.g., impersonal contact, therapeutic relationship, physical presence) |
|  |  | 1.1.6. | Useful in my job: Harder to work with non-verbal behaviour & emotions |
|  |  | 1.2. | Using the system causes me to accomplish tasks more slowly/lowers my productivity |
|  |  | 1.3. | Using online consultations will negatively influence my career |
|  | 2. Effort expectancy | 2.1. | I have difficulties in performing online consultations |
|  |  | 2.1.1. | Online consultations are more exhausting |
|  |  | 2.2. | Learning to use the *technology* for online consultations is hard for me |
|  | 3. Attitude towards using technology | 3.1. | Doing online consultations makes my job less interesting |
|  |  | 3.1.1. | I miss closeness, contact, authentic meeting |
|  |  | 3.2. | I do not like doing online consultations (as compared to face to face) |
|  | 4. Social influence | 4.1. | People that influence my behaviour think I should do online consultations |
|  |  | 4.2. | People that influence my behaviour think I should NOT do online consultations |
|  |  | 4.3. | My organisation/association has not provided sufficient support for online consultations |

Table B.1 – continued

| **Topic** | **Category** | **Nr.** | **Subcategory** |
| --- | --- | --- | --- |
| mental health  professional factors | 5. Facilitating conditions | 5.1. | I do not have the resources (materials, space) to do online consultations |
|  |  | 5.2. | I lack knowledge about (processes specific to) online consultations/I would like more education. |
|  |  | 5.3. | The online consultations software is not compatible with other systems/practices I use |
|  |  | 5.3.1 | It is difficult to maintain good written reports about sessions |
|  |  | 5.4. | I am lacking a person (or group) that is available for assistance with system difficulties |
|  | 6. Anxiety | 6.1. | I feel apprehension about/uncomfortable with doing online consultations |
|  |  | 6.1.1 | fear about loss of privacy by the therapist (e.g., sharing a skype number, patients recording the session) |
|  |  | 6.2. | I am afraid of making mistakes that I cannot correct |
|  |  | 6.2.1 | I am afraid of experiencing technical difficulties |
|  |  | 6.3. | Online consultations are somewhat intimidating to me |
| Client-oriented factors | 7. Performance expectancy | 7 | Clients have questions about whether online consultations are useful for their mental health |
|  | 8. Effort expectancy | 8 | Online consultations cost clients a lot of time and energy |
|  | 9. Social influence | 9.1. | People that influence client's behaviour think they should not do online consultations |
|  |  | 9.2. | Clients are afraid that others will think badly of them if they would use online consultations |
|  | 10. Facilitating conditions | 10.1. | Clients do not have all technical preconditions and the necessary undisturbed quiet space for online consultations |
|  |  | 10.2. | Clients are lacking technical support in case of technical problems |
|  |  | 10.3. | Clients are lacking the *technical* knowledge to use online consultations |
|  | 11. Anxiety | 11 | Client feels apprehension about/uncomfortable with doing online consultations |
|  | 12. Concerns regarding data security | 12 | Clients have concerns about privacy/security |

Table B.1 – continued

| **Topic** | **Category** | **Nr.** | **Subcategory** |
| --- | --- | --- | --- |
| Client-oriented factors | 13. Knowledge | 13 | Clients are lacking knowledge about online consultations |
|  | 14. Attitudes | 14 | Clients are not interested in using online consultations |
| Contextual factors | 15. Contextual factors | 15.1. | Concerns about policy, administration |
|  |  | 15.2 | Concerns about reimbursement & insurance |
|  |  | 15.3 | Concerns about charging and management of payments |
|  |  | 15.4 | Concerns about limits of responsibility & legal aspects |
|  |  | 15.5 | Concerns about unreliable/unstable connectivity, technical difficulties (e.g., internet, lag,..) |
|  |  | 15.6 | Concerns about privacy/security of software (e.g., GDPR) |
|  |  | 15.7 | Price of high-quality online platforms |
|  |  | 15.8 | Ethical concerns |
| Non-specific factors | 16. non-specific factors | 16.1 | I did not or do not have a need for online consultations |
|  |  | 16.2 | no specific reason given for not making use of online consultations in the past |

Table B.2

Reasons for not using online consultations in the past coded according to the UTAUT-based codebook.

|  | **1. Performance Expectancy (PE)** | | | | | | | | | **2. Effort Expectancy (EE)** | | | **3. Attitude (AT)** | | | **4. Social Influence (SI)** | | |
| --- | --- | --- | --- | --- | --- | --- | --- | --- | --- | --- | --- | --- | --- | --- | --- | --- | --- | --- |
| **Country** | **1.1.** | **1.1.1.** | **1.1.2.** | **1.1.3.** | **1.1.4.** | **1.1.5.** | **1.1.6.** | **1.2.** | **1.3.** | **2.1.** | **2.1.1.** | **2.2.** | **3.1.** | **3.1.1.** | **3.2.** | **4.1.** | **4.2.** | **4.3.** |
| Austria (N=65) | 2 | 3 | 3 | 2 | 8 | 13 | 11 | 1 | 0 | 0 | 1 | 0 | 0 | 8 | 1 | 0 | 0 | 0 |
| Belgium (N=249) | 7 | 10 | 13 | 18 | 33 | 42 | 31 | 5 | 0 | 16 | 5 | 2 | 0 | 7 | 0 | 0 | 0 | 0 |
| Cyprus (N=45) | 0 | 2 | 4 | 2 | 3 | 11 | 7 | 0 | 0 | 0 | 0 | 0 | 0 | 2 | 0 | 0 | 0 | 2 |
| France (N=250) | 3 | 3 | 20 | 5 | 16 | 44 | 28 | 0 | 0 | 0 | 2 | 0 | 0 | 0 | 3 | 0 | 0 | 2 |
| Germany (N=166) | 0 | 4 | 2 | 10 | 28 | 33 | 20 | 0 | 0 | 0 | 2 | 0 | 0 | 8 | 0 | 0 | 0 | 0 |
| Italy (N=250) | 6 | 13 | 10 | 14 | 22 | 45 | 24 | 0 | 0 | 2 | 4 | 0 | 1 | 3 | 1 | 0 | 0 | 0 |
| Lebanon (N=73) | 7 | 0 | 4 | 2 | 1 | 12 | 4 | 0 | 0 | 0 | 0 | 0 | 0 | 1 | 0 | 0 | 0 | 0 |
| Lithuania (N=119) | 0 | 5 | 17 | 22 | 16 | 23 | 6 | 0 | 0 | 0 | 2 | 1 | 0 | 0 | 1 | 0 | 0 | 2 |
| Netherlands (N=81) | 8 | 6 | 4 | 12 | 23 | 12 | 21 | 5 | 0 | 4 | 5 | 1 | 0 | 10 | 3 | 0 | 0 | 0 |
| Norway (N=250) | 2 | 10 | 7 | 15 | 33 | 42 | 7 | 0 | 0 | 0 | 1 | 0 | 0 | 5 | 2 | 0 | 0 | 7 |
| Portugal (N=249) | 5 | 10 | 22 | 6 | 21 | 61 | 39 | 0 | 0 | 1 | 5 | 0 | 1 | 13 | 1 | 0 | 0 | 0 |
| Spain (N=31) | 1 | 0 | 0 | 1 | 1 | 0 | 1 | 0 | 0 | 2 | 0 | 0 | 0 | 2 | 0 | 0 | 0 | 1 |
| Sweden (N=250) | 4 | 8 | 5 | 17 | 26 | 19 | 16 | 0 | 1 | 6 | 0 | 1 | 1 | 5 | 0 | 0 | 1 | 2 |
| **Total (N=2078)** | 45 | 74 | 111 | 126 | 231 | 357 | 215 | 11 | 1 | 31 | 27 | 5 | 3 | 64 | 12 | 0 | 1 | 16 |

Table B.2 – continued

|  | **5. Facilitating Conditions (FC)** | | | | | **6. Anxiety (AN)** | | | | | **7. Client factors - PE** | **8. Client factors - EE** | **9. Client factors - SI** | | **10. Client factors - FC** | | | **11. Client factors - AN** |
| --- | --- | --- | --- | --- | --- | --- | --- | --- | --- | --- | --- | --- | --- | --- | --- | --- | --- | --- |
| **Country** | **5.1.** | **5.2.** | **5.3.** | **5.3.1** | **5.4.** | **6.1.** | **6.1.1** | **6.2.** | **6.2.1** | **6.3.** | **7.** | **8.** | **9.1.** | **9.2.** | **10.1.** | **10.2.** | **10.3.** | **11** |
| Austria (N=65) | 1 | 4 | 0 | 0 | 0 | 1 | 2 | 1 | 2 | 0 | 0 | 0 | 0 | 1 | 3 | 0 | 0 | 2 |
| Belgium (N=249) | 15 | 19 | 0 | 1 | 0 | 10 | 10 | 0 | 7 | 2 | 0 | 2 | 0 | 0 | 33 | 1 | 4 | 10 |
| Cyprus (N=45) | 1 | 2 | 0 | 0 | 0 | 0 | 2 | 1 | 3 | 0 | 0 | 0 | 0 | 0 | 3 | 0 | 1 | 0 |
| France (N=250) | 5 | 48 | 0 | 0 | 1 | 2 | 3 | 0 | 2 | 0 | 1 | 0 | 0 | 0 | 24 | 0 | 1 | 4 |
| Germany (N=166) | 1 | 7 | 2 | 0 | 0 | 4 | 7 | 0 | 4 | 0 | 1 | 1 | 0 | 0 | 18 | 0 | 5 | 8 |
| Italy (N=250) | 3 | 7 | 0 | 0 | 0 | 5 | 4 | 0 | 1 | 2 | 5 | 3 | 0 | 0 | 14 | 0 | 3 | 9 |
| Lebanon (N=73) | 2 | 2 | 0 | 0 | 1 | 0 | 4 | 0 | 0 | 0 | 0 | 1 | 0 | 0 | 7 | 0 | 0 | 2 |
| Lithuania (N=119) | 7 | 22 | 2 | 0 | 1 | 0 | 3 | 0 | 1 | 0 | 0 | 0 | 0 | 0 | 19 | 2 | 1 | 1 |
| Netherlands (N=81) | 2 | 4 | 0 | 0 | 1 | 3 | 3 | 0 | 1 | 0 | 2 | 0 | 0 | 0 | 13 | 0 | 5 | 1 |
| Norway (N=250) | 8 | 5 | 0 | 3 | 1 | 1 | 3 | 0 | 9 | 1 | 2 | 0 | 0 | 0 | 23 | 0 | 2 | 3 |
| Portugal (N=249) | 2 | 15 | 6 | 0 | 0 | 4 | 5 | 1 | 2 | 1 | 2 | 0 | 0 | 0 | 29 | 0 | 3 | 6 |
| Spain (N=31) | 2 | 1 | 0 | 0 | 0 | 0 | 0 | 0 | 0 | 0 | 1 | 0 | 0 | 0 | 1 | 0 | 0 | 0 |
| Sweden (N=250) | 11 | 12 | 12 | 1 | 0 | 2 | 2 | 14 | 1 | 0 | 2 | 0 | 0 | 0 | 6 | 1 | 8 | 6 |
| **Total (N=2078)** | 60 | 148 | 22 | 5 | 5 | 32 | 48 | 17 | 33 | 6 | 16 | 7 | 0 | 1 | 193 | 4 | 33 | 52 |

Table B.2 – continued

|  | **12. Client factors - DS** | **13. Client factors - KN** | **14.Client factors - AT** | **15. Contextual factors** | | | | | | | | **16. Non-specific factors** | |
| --- | --- | --- | --- | --- | --- | --- | --- | --- | --- | --- | --- | --- | --- |
| **Country** | **12.** | **13.** | **14.** | **15.1.** | **15.2.** | **15.3.** | **15.4.** | **15.5.** | **15.6** | **15.7** | **15.8** | **16.1.** | **16.2.** |
| Austria (N=65) | 1 | 0 | 4 | 1 | 0 | 5 | 8 | 12 | 28 | 1 | 0 | 0 | 4 |
| Belgium (N=249) | 0 | 1 | 14 | 5 | 3 | 17 | 3 | 42 | 65 | 5 | 7 | 1 | 2 |
| Cyprus (N=45) | 4 | 0 | 0 | 1 | 0 | 5 | 0 | 3 | 7 | 0 | 6 | 0 | 0 |
| France (N=250) | 0 | 2 | 1 | 0 | 2 | 23 | 3 | 33 | 60 | 4 | 2 | 0 | 0 |
| Germany (N=166) | 3 | 0 | 4 | 5 | 5 | 3 | 5 | 29 | 46 | 1 | 3 | 0 | 1 |
| Italy (N=250) | 1 | 1 | 4 | 10 | 0 | 11 | 8 | 16 | 42 | 0 | 1 | 0 | 0 |
| Lebanon (N=73) | 0 | 0 | 1 | 0 | 0 | 10 | 0 | 14 | 10 | 0 | 6 | 0 | 0 |
| Lithuania (N=119) | 0 | 1 | 3 | 8 | 0 | 2 | 6 | 19 | 35 | 0 | 1 | 0 | 0 |
| Netherlands (N=81) | 1 | 0 | 2 | 1 | 0 | 0 | 3 | 12 | 15 | 0 | 3 | 0 | 0 |
| Norway (N=250) | 1 | 0 | 9 | 0 | 0 | 0 | 0 | 34 | 31 | 1 | 1 | 0 | 1 |
| Portugal (N=249) | 2 | 0 | 3 | 4 | 0 | 8 | 9 | 20 | 62 | 1 | 5 | 0 | 0 |
| Spain (N=31) | 1 | 0 | 0 | 0 | 0 | 2 | 1 | 0 | 5 | 0 | 1 | 0 | 0 |
| Sweden (N=250) | 0 | 2 | 2 | 1 | 1 | 2 | 4 | 27 | 36 | 3 | 2 | 1 | 9 |
| **Total (N=2078)** | 14 | 7 | 47 | 36 | 11 | 88 | 50 | 261 | 442 | 16 | 38 | 2 | 17 |

Table B.3

Self-reported concerns regarding online consultations coded according to the UTAUT-based codebook.

|  | **1. Performance Expectancy (PE)** | | | | | | | | | **2. Effort Expectancy (EE)** | | | **3. Attitude (AT)** | | | **4. Social Influence (SI)** | | |
| --- | --- | --- | --- | --- | --- | --- | --- | --- | --- | --- | --- | --- | --- | --- | --- | --- | --- | --- |
| **Country** | **1.1.** | **1.1.1.** | **1.1.2.** | **1.1.3.** | **1.1.4.** | **1.1.5.** | **1.1.6.** | **1.2.** | **1.3.** | **2.1.** | **2.1.1.** | **2.2.** | **3.1.** | **3.1.1.** | **3.2.** | **4.1.** | **4.2.** | **4.3.** |
| Austria (N=65) | 2 | 3 | 3 | 2 | 8 | 13 | 11 | 1 | 0 | 0 | 1 | 0 | 0 | 8 | 1 | 0 | 0 | 0 |
| Belgium (N=249) | 7 | 10 | 13 | 18 | 33 | 42 | 31 | 5 | 0 | 16 | 5 | 2 | 0 | 7 | 0 | 0 | 0 | 0 |
| Cyprus (N=45) | 0 | 2 | 4 | 2 | 3 | 11 | 7 | 0 | 0 | 0 | 0 | 0 | 0 | 2 | 0 | 0 | 0 | 2 |
| France (N=250) | 3 | 3 | 20 | 5 | 16 | 44 | 28 | 0 | 0 | 0 | 2 | 0 | 0 | 0 | 3 | 0 | 0 | 2 |
| Germany (N=166) | 0 | 4 | 2 | 10 | 28 | 33 | 20 | 0 | 0 | 0 | 2 | 0 | 0 | 8 | 0 | 0 | 0 | 0 |
| Italy (N=250) | 6 | 13 | 10 | 14 | 22 | 45 | 24 | 0 | 0 | 2 | 4 | 0 | 1 | 3 | 1 | 0 | 0 | 0 |
| Lebanon (N=73) | 7 | 0 | 4 | 2 | 1 | 12 | 4 | 0 | 0 | 0 | 0 | 0 | 0 | 1 | 0 | 0 | 0 | 0 |
| Lithuania (N=119) | 0 | 5 | 17 | 22 | 16 | 23 | 6 | 0 | 0 | 0 | 2 | 1 | 0 | 0 | 1 | 0 | 0 | 2 |
| Netherlands (N=81) | 8 | 6 | 4 | 12 | 23 | 12 | 21 | 5 | 0 | 4 | 5 | 1 | 0 | 10 | 3 | 0 | 0 | 0 |
| Norway (N=250) | 2 | 10 | 7 | 15 | 33 | 42 | 7 | 0 | 0 | 0 | 1 | 0 | 0 | 5 | 2 | 0 | 0 | 7 |
| Portugal (N=249) | 5 | 10 | 22 | 6 | 21 | 61 | 39 | 0 | 0 | 1 | 5 | 0 | 1 | 13 | 1 | 0 | 0 | 0 |
| Spain (N=31) | 1 | 0 | 0 | 1 | 1 | 0 | 1 | 0 | 0 | 2 | 0 | 0 | 0 | 2 | 0 | 0 | 0 | 1 |
| Sweden (N=250) | 4 | 8 | 5 | 17 | 26 | 19 | 16 | 0 | 1 | 6 | 0 | 1 | 1 | 5 | 0 | 0 | 1 | 2 |
| **Total (N=2078)** | **45** | **74** | **111** | **126** | **231** | **357** | **215** | **11** | **1** | **31** | **27** | **5** | **3** | **64** | **12** | **0** | **1** | **16** |

Table B.3 - continued

|  | **5. Facilitating Conditions (FC)** | | | | | **6. Anxiety (AN)** | | | | | **7. Client factors - PE** | **8. Client factors - EE** | **9. Client factors - SI** | | **10. Client factors - FC** | | | **11. Client factors - AN** |
| --- | --- | --- | --- | --- | --- | --- | --- | --- | --- | --- | --- | --- | --- | --- | --- | --- | --- | --- |
| **Country** | **5.1.** | **5.2.** | **5.3.** | **5.3.1** | **5.4.** | **6.1.** | **6.1.1** | **6.2.** | **6.2.1** | **6.3.** | **7.** | **8.** | **9.1.** | **9.2.** | **10.1.** | **10.2.** | **10.3.** | **11** |
| Austria (N=65) | 1 | 4 | 0 | 0 | 0 | 1 | 2 | 1 | 2 | 0 | 0 | 0 | 0 | 1 | 3 | 0 | 0 | 2 |
| Belgium (N=249) | 15 | 19 | 0 | 1 | 0 | 10 | 10 | 0 | 7 | 2 | 0 | 2 | 0 | 0 | 33 | 1 | 4 | 10 |
| Cyprus (N=45) | 1 | 2 | 0 | 0 | 0 | 0 | 2 | 1 | 3 | 0 | 0 | 0 | 0 | 0 | 3 | 0 | 1 | 0 |
| France (N=250) | 5 | 48 | 0 | 0 | 1 | 2 | 3 | 0 | 2 | 0 | 1 | 0 | 0 | 0 | 24 | 0 | 1 | 4 |
| Germany (N=166) | 1 | 7 | 2 | 0 | 0 | 4 | 7 | 0 | 4 | 0 | 1 | 1 | 0 | 0 | 18 | 0 | 5 | 8 |
| Italy (N=250) | 3 | 7 | 0 | 0 | 0 | 5 | 4 | 0 | 1 | 2 | 5 | 3 | 0 | 0 | 14 | 0 | 3 | 9 |
| Lebanon (N=73) | 2 | 2 | 0 | 0 | 1 | 0 | 4 | 0 | 0 | 0 | 0 | 1 | 0 | 0 | 7 | 0 | 0 | 2 |
| Lithuania (N=119) | 7 | 22 | 2 | 0 | 1 | 0 | 3 | 0 | 1 | 0 | 0 | 0 | 0 | 0 | 19 | 2 | 1 | 1 |
| Netherlands (N=81) | 2 | 4 | 0 | 0 | 1 | 3 | 3 | 0 | 1 | 0 | 2 | 0 | 0 | 0 | 13 | 0 | 5 | 1 |
| Norway (N=250) | 8 | 5 | 0 | 3 | 1 | 1 | 3 | 0 | 9 | 1 | 2 | 0 | 0 | 0 | 23 | 0 | 2 | 3 |
| Portugal (N=249) | 2 | 15 | 6 | 0 | 0 | 4 | 5 | 1 | 2 | 1 | 2 | 0 | 0 | 0 | 29 | 0 | 3 | 6 |
| Spain (N=31) | 2 | 1 | 0 | 0 | 0 | 0 | 0 | 0 | 0 | 0 | 1 | 0 | 0 | 0 | 1 | 0 | 0 | 0 |
| Sweden (N=250) | 11 | 12 | 12 | 1 | 0 | 2 | 2 | 14 | 1 | 0 | 2 | 0 | 0 | 0 | 6 | 1 | 8 | 6 |
| **Total (N=2078)** | **60** | **148** | **22** | **5** | **5** | **32** | **48** | **17** | **33** | **6** | **16** | **7** | **0** | **1** | **193** | **4** | **33** | **52** |

Table B.3 - continued

|  | **12. Client factors - DS** | **13. Client factors - KN** | **14.Client factors - AT** | **15. Contextual factors** | | | | | | | | **16. Non-specific factors** | |
| --- | --- | --- | --- | --- | --- | --- | --- | --- | --- | --- | --- | --- | --- |
| **Country** | **12.** | **13.** | **14.** | **15.1.** | **15.2.** | **15.3.** | **15.4.** | **15.5.** | **15.6** | **15.7** | **15.8** | **16.1.** | **16.2.** |
| Austria (N=65) | 1 | 0 | 4 | 1 | 0 | 5 | 8 | 12 | 28 | 1 | 0 | 0 | 4 |
| Belgium (N=249) | 0 | 1 | 14 | 5 | 3 | 17 | 3 | 42 | 65 | 5 | 7 | 1 | 2 |
| Cyprus (N=45) | 4 | 0 | 0 | 1 | 0 | 5 | 0 | 3 | 7 | 0 | 6 | 0 | 0 |
| France (N=250) | 0 | 2 | 1 | 0 | 2 | 23 | 3 | 33 | 60 | 4 | 2 | 0 | 0 |
| Germany (N=166) | 3 | 0 | 4 | 5 | 5 | 3 | 5 | 29 | 46 | 1 | 3 | 0 | 1 |
| Italy (N=250) | 1 | 1 | 4 | 10 | 0 | 11 | 8 | 16 | 42 | 0 | 1 | 0 | 0 |
| Lebanon (N=73) | 0 | 0 | 1 | 0 | 0 | 10 | 0 | 14 | 10 | 0 | 6 | 0 | 0 |
| Lithuania (N=119) | 0 | 1 | 3 | 8 | 0 | 2 | 6 | 19 | 35 | 0 | 1 | 0 | 0 |
| Netherlands (N=81) | 1 | 0 | 2 | 1 | 0 | 0 | 3 | 12 | 15 | 0 | 3 | 0 | 0 |
| Norway (N=250) | 1 | 0 | 9 | 0 | 0 | 0 | 0 | 34 | 31 | 1 | 1 | 0 | 1 |
| Portugal (N=249) | 2 | 0 | 3 | 4 | 0 | 8 | 9 | 20 | 62 | 1 | 5 | 0 | 0 |
| Spain (N=31) | 1 | 0 | 0 | 0 | 0 | 2 | 1 | 0 | 5 | 0 | 1 | 0 | 0 |
| Sweden (N=250) | 0 | 2 | 2 | 1 | 1 | 2 | 4 | 27 | 36 | 3 | 2 | 1 | 9 |
| **Total (N=2078)** | **14** | **7** | **47** | **36** | **11** | **88** | **50** | **261** | **442** | **16** | **38** | **2** | **17** |
